# Progress in Medicine

**Published:** 1899-12-09

**Authors:** 


### Progress in Medicine.

### RHEUMATISM AND GOUT.

(*Continued from page* 145.)

**Purpura.**—Clifford Beale[12] describes a case of Henoch's purpura in a boy of 11, who presented the classical symptoms of attacks of abdominal pain, swelling about one or two joints, a purpuric rash, and the absence of pyrexia. He refers to the accounts given by Osler and Dusch, showing the frequent presence of hæmorrhages and occasional nephritis or phlebitis. The cases generally end in recovery, but fresh attacks are liable to occur at intervals. An instance of hæmorrhagic purpura in which streptococci were found in the blood by E. Cureton[13] is of interest, since many observers have pointed out the probability of a bacterial infection in this disease. The patient, a boy of 11, suffered from mild purpura for three weeks, when hæmorrhages with pyrexia and delirium set in, and a diminution of red corpuscles to even 310,000 per c.c.m. took place shortly before his death. Whether this was a secondary infection or not is uncertain, but Letzerich's bacillus does not appear to have been detected. Rheumatism of the sterno-xiphoid articulation occasionally appears without the affection of any other joints, and is difficult to diagnose. Hertz and Roustan[14] remark that it may be overlooked, and the pain referred to stomach disorders. They recommend blisters, and salicylates internally. Synovitis of both knees, either together or a little later in one than in the other, is found in patients suffering from interstitial keratitis. The affection is doubtless syphilitic, but it differs from most forms of syphilitic joint disease, in that it is a pure chronic hypersecretion. J. W. Stokes[15] observed three cases in 30 patients suffering from keratitis. There appears to be no pain, redness, or fixation of the joint, and the condition lasts for several months. The value of chloride of calcium in hæmophilia, where the coagulation time of the blood is long, was recognised by A. E. Wright some years ago. G. Mansel Sympson[16] has given it to one patient for the greater part of six years, and considers that the subcutaneous hæmorrhages were lessened by it, and that when wounds occurred the clots were firmer and the bleeding less, if full doses were given. Bone marrow had no effect on the bleeding, but it brought the patient round quickly after severe attacks.

**Gonorrhœal Arthritis.**—The discovery of the gonococcus, not only in diseased joints but also in the heart and blood, shows the general character of the infection, hence the rule that the affection is a mono-articular one is not absolute. G. Still mentions that in children many joints are often attacked together, and Mühsam[17] found in forty cases that ten showed multiple lesions. Less than half recovered complete use of the joints, the treatment which gave best results being that of complete immobilisation of the joint, which was in some instances followed by puncture and irrigation.

**Arthritis Deformans.**—When the hip joint only is attacked in old people the diagnosis must be made from impacted fracture, coxa vara, chronic rheumatism, and conceivably from chronic hydrarthrosis and tuberculous coxitis. C. G. Cumston[18] points out that in tuberculous disease there will be abduction and outward rotation, or adduction and inward rotation, with often some lung affection. In coxa vara the patients are usually young, and adduction and outward rotation are generally present. Hydrarthrosis will show fluctuation, pain, and some restriction of rotation. Chronic rheumatism usually affects more than one joint, there is no shortening, and though some apparent grating sounds may be felt there is no destruction of cartilage. On the other hand, in true arthritis deformans there is pain and true crackling, with some shortening as the disease advances, and restriction of movements, more especially those of abduction and inward rotation. The prognosis is bad. In early stages hot douches with massage may be of use. If the pains in later stages are relieved by immobilisation, massage and passive movements should be used afterwards. Tonics and warm climates are of some value, but surgical measures which give good results in other joints are not desirable at the hip in patients of such advanced age.

**Angioneurotic Œdema.**—T. Stacey Wilson[19] records a case of transient general œdema, commencing suddenly in the feet and ascending the legs. The face and arms then became enormously swollen, but the legs began to grow smaller before the arms reached their full size. In four days it had almost entirely disappeared. There was no affection of the heart present, nor any albumen or casts in the urine, nor any inflammation of the skin. H. B. Baruch[20] notices in localised transient œdema the recurrence and periodicity of the attacks. Cold, overwork, or gastric troubles will bring them on. The swellings vary in diameter from a quarter of an inch to half a foot or more, and most often occur on the face or the extremities. There may be some rise of temperature in the attacks and a sense of burning or tingling in the swellings, which disappear more or less rapidly after attaining their maximum. Treatment is unsatisfactory, but pilocarpine is of some little value, and a dose of calomel seems to cut short certain cases depending upon gastric troubles.

[12] Practitioner, Mar. [13] Lancet, Feb. 25. [14] Brit. Med. Jour., July 29. [15] Med. Quar., April. [16] Lancet, May 13. [17] Med. Rec. [18] Internat. Med. Mag., Aug. [19] Birmingham Med. Jour., April. [20] Med. Rec., Aug. 19.

